# Clinical and Microbiological Evaluation of Brazilian Red Propolis Containing-Dentifrice in Orthodontic Patients: A Randomized Clinical Trial

**DOI:** 10.1155/2020/8532701

**Published:** 2020-01-21

**Authors:** João Hildo de Carvalho Furtado Júnior, Lídia Audrey Rocha Valadas, Said Gonçalves da Cruz Fonseca, Patrícia Leal Dantas Lobo, Lays Helena Maia Calixto, Ana Gleyce Ferreira Lima, Maria Helena Ramos de Aguiar, Isadora Sousa Arruda, Mara Assef Leitão Lotif, Edilson Martins Rodrigues Neto, Marta Maria de França Fonteles

**Affiliations:** ^1^Postgraduate Program in Development and Technological Innovation in Drugs, School of Pharmacy, Federal University of Ceara, Fortaleza, Brazil; ^2^Pharmacy, Dentistry and Nursing College, Federal University of Ceara, Fortaleza, Brazil

## Abstract

**Aim:**

To evaluate the efficacy of dentifrice containing brazilian red propolis (BRP) in adolescents under orthodontic treatment. *Materials and Methods*. This is a randomized, double-blind, clinical trial. A total of 92 participants free from caries were randomized into 2 groups; the first received fluoride dentifrice, and the second received fluoride dentifrice incorporated with BRP. The gingival bleeding index (GBI) was recorded, and saliva was collected on the baseline (D0) and after 28 days (D28) for microbiological analysis. Data from GBI and Colony Forming Units (CFU) (log_10_) were expressed as mean and standard deviation.

**Results:**

The two groups reduced GBI significantly, with no difference in the intergroup analysis. In the intragroup analysis, it was observed that G2 (*p* < 0.001) had a significant reduction for Gram-negative bacteria, while there was significance (*p* < 0.001) had a significant reduction for Gram-negative bacteria, while there was significance (*S. mutans* bacteria, it was observed that only G2 had a statistically significant reduction (*p* < 0.001) had a significant reduction for Gram-negative bacteria, while there was significance (*p* < 0.001) had a significant reduction for Gram-negative bacteria, while there was significance (

**Conclusions:**

Dentifrice containing BRP demonstrated better clinical and microbiological activity. Future studies are needed to better identify effects to establish the use of dentifrice containing propolis in biofilm control.

## 1. Introduction

The oral cavity aggregates diverse communities of microorganisms which reside on various surfaces as a biofilm. Usually, these virus and bacteria communities coexist in balance. Oral health is directly related to this balance, where a balanced diet and good oral hygiene are the main factors for this maintenance. However, when the oral environment undergoes changes, this ecosystem is unbalanced, thus resulting in changes between the microorganisms and biofilm, thereby increasing the risk of dysbiosis [1].

Gingivitis is considered an inflammatory disease of microbiological origin. Dental caries is defined as dysbiosis caused by frequent exposure of sugars, resulting in demineralized dental tissues. There is consensus that dental biofilm is the major biological determinant for the development of both diseases [2, 3].

The use of brackets due to orthodontic treatment is one of the factors that favors dental biofilm retention due to the difficulty of hygiene and plaque accumulation, and so changes in pH and the development of caries lesions and gingivitis are frequent [4]. The use of orthodontic appliances also increases retention of cariogenic bacteria favoring their growth and development, thus causing an imbalance in the oral microbiota and demineralization around the brackets. It is known that *Streptococcus mutans* (*S. mutans*) is one of the main organisms involved in cariogenic biofilm and have an important virulent role in caries pathogenesis due to its acidogenic and acidic properties [5, 6].

Biofilm develops on the dental surface and is composed of different species of microorganisms, initially formed by Gram-positive and aerobic bacteria, but later on there is sequential colonization of Gram-negative and anaerobic microorganisms. The marginal gingival tissue begins to develop an inflammatory response caused by the toxins released from the microorganisms present in the biofilm. This response occurs when substances are released into the body, such as histamine, in addition to producing inflammatory substances which increase the permeability of blood vessels. Prolonged maintenance of the inflammatory process in the tissues is called chronic inflammation and can promote bone destruction and bone loss. Thus, it is necessary that there is a constant and effective disorganization of this biofilm to prevent these pathological conditions [6].

One strategy to aid in mechanically removing plaque in these patients is through adding an antimicrobial agent with antiplate action in dental formulations, especially dentifrices. These agents ideally have antimicrobial activity to assist in plaque control and inflammation of the gums and anticaries action to prevent or reduce frequent demineralization around the brackets. In addition, it is appropriate for the product to have a good taste and not cause side effects [7].

One of the complementary methods used as an indicator to verify changes in the microbiota is through saliva [8]. Saliva is a biological material that is easy to collect and noninvasive, which can identify different genera and species as it comes into contact with all surfaces of the oral cavity such as the teeth, gums, and tongue. Changes in the oral microbiota can be monitored through salivary samples. Therefore, saliva can identify more microorganisms than the dental biofilm [9, 10].

Nowadays, the incorporation of natural products to dental care is common. Propolis is a complex, nontoxic resinous mixture collected from plant exudates by *Apis mellifera*, in which its biological properties are related to geographical locations and botanical origin [11].

The pharmacological benefits of propolis have been widely explored in various fields of medicine as an important resource for preventing and treating oral and systemic diseases. There are some types of propolis classified according to their physicochemical properties and geographic origin, and among these Brazilian red propolis (BRP) stands out. BRP is the 13^th^ type, and its red coloration is due to the plant's pigments [11, 12].

This type is relatively new and has drawn attention because of its promising chemical composition and pharmacological properties, especially antimicrobial and anti-inflammatory properties. It is found in the Brazilian northeast, a region rich in biodiversity, mainly on the coast in the state of Alagoas in Marechal Deodoro city, and its botanical origin is *Dalbergia ecastophyllum*. This type is unique to this region and possesses isoflavonoids in its composition and led to the National Institute of Industrial Property (INPI), granting the title of Geographical Indication of the region, assuring the international certificate to this city of being the only producer of this type of propolis in the world, with quality independent of time and climate [11–15].

This product has a high medicinal, historical, and economic value. In recent years, BRP has drawn interest from the pharmaceutical industry and its commercial production increased considerably in several countries such as Brazil, Japan, China, Russia, Germany, and France. Therefore, the product is expanding both nationally and internationally. Several studies have been conducted on propolis in several areas such as Medicine, Dentistry, and Chemistry. In Dentistry, studies point to promising results in Endodontics, Cariology, Surgery, Preventive Dentistry, and Periodontics [16].

Both caries and gingivitis diseases can progress slowly throughout life and can be controlled through noninvasive interventions [17]. Dentifrices with bioactive molecules have been studied in *in vitro* research as antimicrobial agents. No reports of the use of dentifrice incorporated with BRP were found in the literature, thus an application for an invention patent was deposited under protocol BR1020170110974. As a consequence of the above, it would have good use in orthodontic patients for the purpose of plaque control through possible antimicrobial activity of this dentifrice. Therefore, the objective of this research was to clinically and microbiologically evaluate the efficacy of a dentifrice incorporated with BRP extract in adolescents with signs of gingivitis.

## 2. Materials and Methods

### 2.1. Study Design and Location

This is a longitudinal, parallel, randomized, double-blind controlled clinical trial and adhered to the CONSORT checklist. The rules of the Strengthening the Reporting of Observational Studies in Epidemiology (STROBE) were followed in order to improve the study methodology. The clinical phase occurred in the city of Aracati-CE, a city where only 0.8% of the population has public fluoridated water coverage [18].

### 2.2. Ethical Aspects, Population, and Sampling

This study was approved by the Ethics Committee of the Federal University of Ceara (approval number 1.552.749), according to resolution no. 466/12 of research involving human beings and with the Declaration of Helsinki under ethical principles for medical research involving human beings.

The sample was designed to demonstrate the statistical superiority of the dentifrice containing red propolis extract compared with the common dentifrice in treating gingivitis, considering a power of 90% (*β* = 0.10) and a significance level of 5% (*α* = 0.05), based on the gingival bleeding index (GBI) measured at the end of the treatment, which was defined as the primary outcome. For this, it was established that the minimum difference between the effects of the two treatments to be detected would correspond to 0.12 points in the GBI, considering a standard deviation of this variable estimated at 0.16 points. This difference represents a reduction of around 32% in the GBI, according to previous studies. In addition, it was defined that the allocation rate would be 1, meaning that the groups would have equal sizes. Thus, using the proper expression for studies of statistical superiority and considering that the primary outcome is a quantitative variable, the sample size needed to satisfy the abovementioned requirements was calculated as being 38 subjects in each group. However, 20% was added to this value in order to cover possible follow-up losses, so that the final sample size was estimated as 46 patients in each group.

An active search was conducted in public elementary and middle schools for the selection of participants. After those responsible for the participants signed the informed consent form and the participants gave consent, 92 adolescents aged 12 to 16 years of both genders, free from caries (ICDAS II = 0), users of fixed orthodontic appliances and having visible plaque index were selected.

Adolescents with a history of allergies such as asthma, urticaria, rhinitis, sinusitis, or intraoral soft tissue injury were excluded from the study. None of the participants underwent antibiotic treatment up to 3 months prior to initiating the study, nor during the course of this clinical trial.

### 2.3. BRP Extract and Dentrifice Preparation

The BRP extract was collected from the city of Marechal Deodoro (South Latitude 9°44.555′, West Latitude 35°52.080′, and altitude of 18.1 m above sea level), a region with geographical indication granted by the National Institute of Industrial Property, in the state of Alagoas, Brazil. First, 150 grams of the red propolis extract was taken and extracted with 1 L of cereal alcohol of 96° graduation and then diluted to a concentration of 1%. The BRP extract at 1% concentration (previously studied antimicrobial concentration) was incorporated into the fluoridated dentifrice (1500 ppm) in the Pharmaceutics laboratory of the Pharmacy course of theFederal University of Ceara, Brazil. Dentifrices were formulated with the same taste, color, and odor after chemical identification of the constituents by High Performance Liquid Chromatography (HPLC) with the main constituents of Quercetin, Vestitol, and Neovestitol being identified. Identification was performed by comparing the chromatographic profile of the BRP samples in relation to the standards of the isolated chemical constituents subjected to the same analysis conditions. Thus, when there was a coincidence between retention times, the UV absorption spectrum was compared between the sample and reference, seeking to establish similarity.

### 2.4. Treatment Application

The participants were randomly distributed into two groups: Control Group (G1), Commercial Fluoride dentifrice (1500 ppm MFP) and Test Group (G2), Fluoride dentifrice with BRP (1500 ppm MFP). There were 46 participants in both G1 and the Control Group. Saliva collection was performed for microbiological analysis and the Gingival Bleeding Index (GBI) using the WHO periodontal probe and buccal mirror before starting treatment (D0) and on the last day (D28).

The dentifrices were stored in equal tubes to keep the applied treatment type confidential to both the researchers involved in the clinical trial and the participants, thus guaranteeing a double-blind study. All participants received a toothbrush of the same brand with a straight handle, small head and soft bristles, and the treatment toothpaste. Standardized oral hygiene instruction was conducted through a single instructor for all participants. All participants received the recommendations to be followed in writing to reinforce the instructions.

### 2.5. Saliva Collection and Microbiological Analysis

Each patient initially chewed one piece of a 3 × 3 cm plastic film (Parafilm®) for 60 s to stimulate saliva production and release the bacteria from the dental biofilm. Saliva was collected using a plastic device and stored in sterile microcentrifuge tubes (Eppendorf®), which were stored in a polystyrene box containing ice and analyzed in the microbiology laboratory within 2 hours of collection.

The saliva from each patient was collected in two moments (baseline and 28 days after starting treatment). Participants were instructed not to eat or drink at least 2 hours before saliva collection, and the samples were collected under the same conditions operated between 9 : 00 and 11 : 00 a.m, so that the circadian influence was minimized.

A volume of 0.1 mL of each sample was transferred to a sterile hemolysis tube containing0.9 mL of saline. This procedure was repeated twice, establishing dilutions of 1 : 10 and 1 : 100. A volume corresponding to 10 *μ*L of each dilution was seeded in Agar mitis bacitracin (MSB) and MacConkey Agar in triplicate for evidence of *S. mutans* and Gram-negative bacteria, respectively.

The plates were incubated at 37°C for 48 hours in microaerophilic jars and placed in an oven. Colonies with Gram-morphological characteristics were then counted after this period. Bacteria were expressed as CFU/mL of saliva.

### 2.6. Clinical Analysis

The patients were submitted to the gingival bleeding index (GBI) test on all teeth by a single examiner. The mesial, buccal, distal, and lingual surfaces were evaluated. The presence of gingival bleeding was evaluated before treatment started (D0) and 4 weeks later (D28) via a WHO probe.

### 2.7. Statistical Analysis

Clinical data were expressed as absolute and percentage frequency and compared using the chi-square test. The GBI and CFU (log10) data were expressed as mean and standard deviation, submitted to the Kolmogorov–Smirnov normality test and compared using the Wilcoxon and Mann–Whitney tests (SPSS v.20.0; *p* < 0.05).

## 3. Results

**Figure 1:**
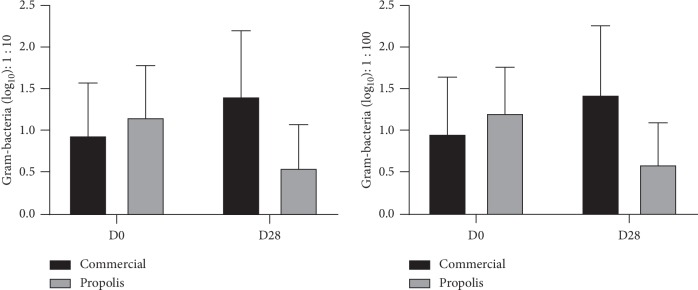
Salivary reduction of colony-forming units (CFU) log_10_ of gram-negative bacteria at the 1 : 10/1 : 100 dilutions.

**Figure 2:**
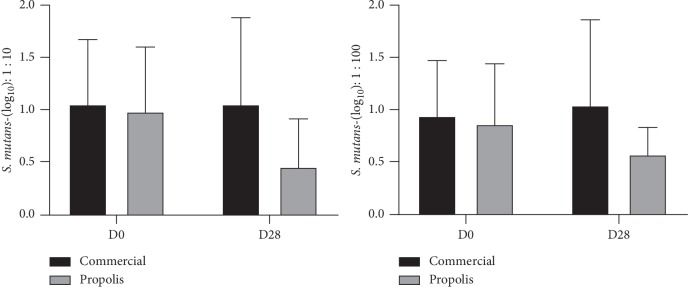
Salivary reduction of colony-forming units (CFU) log_10_ of *S. mutans* bacteria at the 1 : 10/1 : 100 dilutions.

Table 1 shows the analysis of Gram-negative and S. mutans CFU in the groups treated for 28 days with fluoride dentifrice (G1) and BRP-containing fluoride dentifrice (G2). The assay was performed using two different dilutions, 1 : 10 and 1 : 100–for colony counting, revealing concordant results. In the intragroup analysis, from D0 to D28 G1 presents a statistically significant increase of CFU for both Gram-negative bacteria and S. mutans, while for G2, a significant reduction of this parameter occurred. The intergroup analysis (G1 × G2) revealed, at D28, also a significant decrease of G2 when compared with G1, for both microorganisms assayed (Figures 1 and 2).

Table 2 shows the gingival bleeding index (GBI) and CFU for Gram-negative bacteria and S. mutans in the groups treated with fluoride dentifrice (G1) or with BRP-containing fluoride dentifrice (G2) at D0 and D28. The two groups showed a statistically significant GBI reduction from D0 to D28, with no intergroup difference. Similarly, to the results displayed in Table 1, for the Gram-negative bacteria counts, it was observed a significant intragroup increase for G1 (*p*=0.003) and for G2, a significant reduction (*p* < 0.001); also, in the intergroup analysis, the value for G2 was significantly lower (*p* < 0.001) than G1. For S. mutans, only G2 showed a significant reduction (*p* < 0.001) in the intragroup analysis (D0 × D28), and when compared with G1, there was significance decrease (*p*=0.006).

## 4. Discussion

This study evaluated the efficacy of a new toothpaste incorporated with BRP for the biofilm control in orthodontic patients, obtaining efficacy after 4 weeks of treatment. Resistance to synthetic antimicrobials and the search for substances with pharmacological properties with lower adverse effects have caused an increased interest in natural products. The high demand for propolis and the modernization of analytical devices have contributed to launch several products in the market [19–22].

Propolis is distinguished by the broad Gram-positive and Gram-negative antimicrobial spectrum against colonizers of the oral biofilm such as *S. mutans*, *Lactobacillus*, *P. gingivalis*, *Actinomyces naeslundii*, *Aggregatibacter actinomycetemcomitans*, *Porphyromonas gingivalis*, and *Prevotella intermedia* [14, 20]. In this study, BRP toothpaste demonstrated efficacy in gingivitis control and bacteria reduction as cited in the literature.

BRP has potent antimicrobial activity even at the concentration of 0.1%, with antimicrobial and anti-inflammatory activity proven *in vitro* and *in vivo*. This is due to the high concentration of flavonoids and phenolic compounds [14, 16]. The group treated with propolis at 1% concentration in this study presented better clinical and microbiological results when compared with commercial toothpaste. Several studies have reported the antimicrobial and anti-inflamatory effects of BRP, but this study is the first to analyze the properties of BRP in a toothpaste.

Caries and periodontal disease are the main oral diseases, in which dental biofilm is one of the main biological determinants that is common for the development of both diseases. Several factors can modulate these diseases, especially the quality of oral hygiene and eating habits. In addition, it is known that the use of fixed appliances facilitates areas of dental plaque stagnation, increasing the susceptibility of demineralization around brackets and gingivitis [23, 24].

Cagetti et al. [25] report that gingivitis is the most prevalent type of periodontal disease in adolescents and that it increases with frequency and severity at puberty. In a randomized double-blind clinical trial, the authors compared the effects of a fluoride dentifrice (control group) with a dentifrice-containing fluoride, triclosan, cetylpyridinium chloride, and essential oils (test group) for plaque control and gingival inflammation after four weeks of use. The dentifrice test showed better anti-plaque results, showing that bioactive molecules is a good strategy to control biofilm.

New strategies for improving toothpaste and microbiota control are being developed every day; for example, Adams et al. [26] conducted a clinical trial in which they evaluated a dentifrice with enzymes and proteins for controlling supragengival plaque for four weeks, where the dentifrice test when compared with a fluoridated dentifrice control had better results.

Streptococcus is one of the most common microorganisms found in the oral cavity, being the pioneer species after dental eruption. Species such as *Veillonella*, *Haemophilus*, *Neisseria* spp., *Prevotella*, and *Fusobacterium* are normally found in dental plaque, on the tongue and in saliva, and oral diseases can develop when this balance is broken. The results showed that the BRP dentifrice presented a statistically significant reduction at the end of the treatment (D28) for *S. mutans* (*p* < 0.001) and Gram-negative bacteria (*p* < 0.001). The group treated with fluoridated dentifrice showed an increase in bacteria in the salivary findings without S. mutans (*p*=0.612) and statistically significant for Gram-negative bacteria (*p*=0.003).

Gingivitis and periodontitis may be prevented by controlling supragingival biofilm. However, situations such as the use of orthodontic appliances may require using a product which improves the mechanical removal of the biofilm [27]. All participants had gingivitis and gingival inflammation at the beginning of the study. At the end of the clinical trial, both groups had a reduction in the gingival bleeding index (GBI), fluoride dentifrice (*p*=0.01), and BRP dentifrice (*p* < 0.001), and there was no statistical difference in the intergroup analysis (*p*=0.135).

Figuero et al. [2] performed a systematic review on the effect of mechanical and chemical plaque control in the fight against oral diseases such as caries and gingivitis. The authors reported that fluoride addition is significant for caries, while antimicrobials are significant for gingivitis. In addition, motivational programs and supervised brushing show a significant effect on plaque reduction, and this explains the reduction in the GBI in both groups of the present study because all participants had educational brushing activities prior to starting the clinical trial. According to Herrera et al. [7], mechanical control is essential in the prevention of caries and periodontal disease, once again reinforcing the findings of the present study.

Dental biofilm removal, oral hygiene practices, and professional intervention are essential to eliminate dental biofilm, and retentive factors are the critical elements in treating gingivitis [28]. Mechanical removal of plaque through brushing contributes to maintaining gingival health, but there is great evidence that the use of antimicrobial substances besides brushing contributes to plaque control and prevents gingivitis in situations of greater risk [25].

The etiologies of dental caries and periodontal diseases are independent. However, some factors are common to both diseases, such as the presence of biofilm [3]. The present dentifrice reduced bacteria related to both diseases, as well as being fluoridated (1500 ppm F), and therefore constitutes a strategy for preventing and controlling both diseases.

Although there is no consensus in the literature, data suggest that the association of fluoridated dentifrice with antimicrobial agents in patients with retention such as in orthodontic appliances may be more effective than fluoride dentifrice used alone [29]. In the intergroup comparison of the present study, the propolis dentifrice had superior results when compared with the fluoride dentifrice in relation to reducing *S. mutans* (*p*=0.006) and Gram-negative bacteria (*p* < 0.001).

## 5. Conclusions

In conclusion, the BRP dentifrice demonstrated better antimicrobial activity against *S. mutans*, Gram-negative bacteria, and in reducing the marginal bleeding index during the treatment period. Future studies are needed to better identify effects to establish the use of dentifrice in the control of dental biofilm.

## Figures and Tables

**Table 1:** Reduction in the number of colony-forming units (CFU) of Gram-negative bacteria and *S. mutans* in the saliva samples verified on the groups.

Toothpaste		D0	D28	*p* value^a^	Δ	*p* value^b^
Dilution 1 : 10						
Gram	G1	0.95 ± 0.63	1.42 ± 0.79	0.001	0.47 ± 0.78	<0.001
	G2	1.17 ± 0.62	0.56 ± 0.52	<0.001	−0.60 ± 0.54	
*S. mutans*	G1	1.06 ± 0.62	1.06 ± 0.83	0.869	0.01 ± 0.73	0.001
	G2	0.99 ± 0.62	0.46 ± 0.46	<0.001	−0.54 ± 0.58	
Dilution 1 : 100						
Gram	G1	0.97 ± 0.68	1.44 ± 0.83	0.005	0.46 ± 0.88	<0.001
	G2	1.22 ± 0.55	0.60 ± 0.50	<0.001	−0.61 ± 0.55	
*S. mutans*	G1	0.95 ± 0.53	1.05 ± 0.82	0.421	−0.02 ± 0.23	0.019
	G2	0.87 ± 0.58	0.58 ± 0.26	0.002	0.12 ± 0.33	

^a^Wilxocon test; ^b^Mann–Whitney test (mean ± DP). Measured as CFU (log_10_).

**Table 2:** Changes in GBI and CFU before and after treatment with different dentifrices.

	Group	D0	D28	*p* value^a^	Δ	*p* value^b^
GBI	G1	38.35 ± 19.39	27.24 ± 14.40	0.001	−11.11 ± 19.88	0.135
	G2	37.94 ± 18.68	20.59 ± 16.45	<0.001	−17.35 ± 12.77	
Gram	G1	3.05 ± 0.66	3.50 ± 0.77	0.003	+0.45 ± 0.84	<0.001
	G2	3.27 ± 0.54	2.66 ± 0.49	<0.001	−0.61 ± 0.54	
*S. mutans*	G1	3.03 ± 0.51	3.09 ± 0.81	0.612	−0.06 ± 0.69	0.006
	G2	2.95 ± 0.57	2.62 ± 0.28	<0.001	−0.33 ± 0.50	

Wilxocon test^a^; Mann–Whitney test^b^. Data are expressed in CFU (log_10_) as mean ± SD.

## Data Availability

The data used to support the findings of this study have been deposited in the Federal University Federal repository ((http://repositorio.ufc.br/bitstream/riufc/40449/1/2019_tese_jhcfurtadojunior.pdf)).

## Supplementary Materials

Supplementary Materials The chromatography graphs.
